# Start Right to End Right: Authentic Open Reading Frame Selection Matters for Nonsense-Mediated Decay Target Identification

**DOI:** 10.3390/genes16111297

**Published:** 2025-11-01

**Authors:** Mojtaba Bagherian, Georgina Harris, Pratosh Sathishkumar, James P. B. Lloyd

**Affiliations:** 1 Australian Research Council Centre of Excellence in Plant Energy Biology, School of Molecular Sciences, University of Western Australia, Crawley 6009, Australia; 2 Australian Research Council Centre of Excellence in Plants for Space, School of Molecular Sciences, University of Western Australia, Crawley 6009, Australia

**Keywords:** genomes, genomics, ORF, genome annotation, NMD, alternative splicing

## Abstract

Backgrounds: Accurate annotation of open reading frames (ORFs) is fundamental for understanding gene function and post-transcriptional regulation. A critical but often overlooked aspect of transcriptome annotation is the selection of authentic translation start sites. Many genome annotation pipelines identify the longest possible ORF in alternatively spliced transcripts, using internal methionine codons as putative start sites. However, this computational approach ignores the biological reality that ribosomes select start codons based on sequence context, not ORF length. Methods: Here, we demonstrate that this practice leads to systematic misannotation of nonsense-mediated decay (NMD) targets in the *Arabidopsis thaliana* Araport11 reference transcriptome. Using TranSuite software to identify authentic start codons, we reanalyzed transcriptomic data from an NMD-deficient mutant. Results: We found that correct ORF annotation more than doubles the number of identifiable NMD targets with premature termination codons followed by downstream exon junctions, from 203 to 426 transcripts. Furthermore, we show that incorrect ORF annotations can lead to erroneous protein structure predictions, potentially introducing computational artefacts into protein databases. Conclusions: Our findings underscore the importance of biologically informed ORF annotation for accurate assessment of post-transcriptional regulation and proteome prediction, with implications for all eukaryotic genome annotation projects.

## 1. Introduction

Genome sequencing is becoming cheaper and easier, with telomere-to-telomere assemblies becoming routine with third-generation sequencing technologies [1,2]. However, annotating transcript models of genomes remains a challenge. With RNA-seq, accurate exon-intron boundaries can be determined and whole transcript models can be generated. Multiple transcript isoforms at a genetic locus can be generated via alternative splicing (AS) to increase the transcriptome diversity. Translated regions of a transcript have been defined as translons [3], but experimentally validating all of the regions of a transcript that are translated by a ribosome is challenging. Instead, software designed to find an open reading frame (ORF) is used to identify regions that can be translated [3]. Many transcripts have a long “main” ORF defined [4], which encodes for a long protein that defines the function of the gene, such as encoding for an enzyme of transcription factor. Accurate detection of an ORF that will be translated (a translon) is integral to predicting the proteins made from a given genome. In many cases, scanning each transcript and finding the longest distance between a start codon and a stop codon will reveal the main ORF that encodes a full-length protein. This approach overcomes the issues caused by short upstream ORFs in the 5′ untranslated region (UTR), which often have regulatory roles in gene expression [4]. Annotating the longest ORF per transcript is how some genome projects, such as the Araport11 transcriptome of the model plant *A. thaliana* was performed [5]. However, AS is known to introduce early stop codons and disrupt the long continuous main ORF. If this premature termination codon (PTC) is introduced late in the coding sequence, the longest predicted ORF would use the same start codon as the transcript isoform without the PTC but would have a short C-terminal truncation. However, if the splicing occurs early in the transcript, the original start codon would only lead to very short ORF; often a longer downstream ORF initiating from an internal methionine would be computationally identified. This computationally predicted longest ORF is unlikely to be a translon, that is, it would not be translated by the ribosome, which would begin translation at the original start codon identified in the transcript without a PTC present [6,7,8]. This mis-annotation of the longest ORF, rather than the translon, using the authentic start codon, would lead to wrongly predicted protein sequences and therefore incorrect protein structures. Furthermore, many PTCs introduced by AS lead to the degradation of the transcript by an evolutionarily conserved eukaryotic quality control pathway [9,10], known as nonsense-mediated mRNA decay (NMD) [11]. AS-coupled to NMD (AS-NMD) has been reported in animals [12,13,14], plants [7,15,16], and fungi [17]. AS-NMD is not simply the process of NMD degrading the products of noisy mis-splicing, but many of the AS events are highly conserved between species [17,18]. These conserved splicing events that introduce PTCs are important for the auto- and cross-regulation of many splicing factors, creating a complex network of post-transcriptional control of splicing factor protein levels via AS-NMD [12,13,14]. The sequences around these AS-NMD splicing events are highly conserved [18,19], and if the AS-NMD was lost during the course of evolution, it would then be reacquired in the new copy of the gene [17]. Therefore, the regulation of splicing factor level via AS-NMD is an important process to study in health, disease, and through evolution [12,13].

How an early stop codon is differentiated from a “normal” stop codon has been the focus of much research in the field. A commonly reported signal that differentiates a PTC from a normal stop codon is the presence of an exon junction ≥ 50–55 nucleotides (nt) downstream of the stop codon, known as the 50–55 nt rule, or exon junction complex (EJC) model of NMD. In the EJC model, the deposition of a protein complex near an exon junction (EJ) also recruits NMD factors, such as UPF3 [11], and when an EJ is downstream of the stop codon (dEJ), then the EJC with NMD factors can trigger NMD at the terminating ribosome [20,21]. The presence of a long 3′ UTR (faux 3′ UTR model) is another suggested elicitor of NMD [11,22,23,24]. The distance of the stop codon from the poly(A) tail (a longer 3′ UTR) [25,26], allowing for more UPF1-binding [27], has been suggested as molecular rulers to measure the length of a 3′ UTR and determine if the transcript is a target of NMD via competition between UPF1 and the poly(A)-binding protein for the eukaryotic release factor during ribosome termination. Studying NMD kinetics at the single-molecule level in human cells has revealed that the exonic sequence downstream of the PTC, PTC to dEJ distance, and number of splice introns after the stop codon all influence NMD efficiency on target transcripts [28].

However, to accurately predict whether a transcript might be an NMD target, not only do we need accurate RNA sequence information for the transcript model (transcriptional start/end sites and exon-intron boundaries), but we also need to know the correct main translon, i.e., the translated ORF. An accurate 3′ UTR annotation allows for accurate prediction of NMD-inducing features [7]. However, in some genome annotation projects, the algorithms used to find the open reading frame (ORF) within the transcript models simply identifies the longest ORF present in a given transcript. Past publications have highlighted the importance of selecting the biologically authentic ORF rather than simply the longest ORF and the potential consequences [6,7,8], and yet issues still exist (see Results) in extant genome annotations. Here we outline why this is a problem for NMD research and protein structural prediction, and then suggest a simple solution using freely available software to re-annotate transcriptomes [8].

In this study, we used the published TranSuite software (https://github.com/anonconda/TranSuite (accessed on 15 May 2025)) [8], with modifications (https://github.com/mojtabagherian/TranSuite (accessed on 10 June 2025)), to re-annotate the ORFs of Araport11. TranSuite does not look at the longest ORF at per transcript level but instead groups transcripts together at the gene level and finds the longest protein from across these transcript isoforms [8]. The start codon responsible for this longest protein is then used to predict the main translon (translated ORF) of each transcript arising from this gene. We show that this helps identify the NMD triggering features (long 3′ UTR and dEJ) of known NMD targets in the model plant *A. thaliana*, and suggest that this improves prediction of the correct protein sequence to use for structural predictions.

## 2. Materials and Methods

Genomes: *A. thaliana* TAIR10 genome (fasta) with Araport11 [5] transcriptome (GFF3) downloaded from Phytozome. The GFF3 was converted to GTF by the GFFread utility in Cufflinks (version 2.2.1) [29]. This was also used to create a transcriptome fasta file from the GTF and genome fasta file:

gffread -w Athaliana_transcripts.fa -g Athaliana_447_TAIR10.fa Athaliana_447_Araport11.gtf

The transcriptome fasta file was converted to a Salmon (version 1.10.0) index, with kmer length set to 31, and Salmon quant was used to quant transcript abundances [30]. The following settings were used:

salmon quant -i A_thaliana_Araport11 -l A -r file.fastq.gz -p 16 --validateMappings --fldMean 150 --numBootstraps 100 --seqBias --gcBias -o output

Previously published RNA-seq data collected from wild-type and *upf1-1 upf3-1* double mutant plants, which have reduced NMD activity [31], were quantified by Salmon [30], as above. Differential transcript expression was assessed through the R (version 4.4.2) package Sleuth (version 0.30.1) [32]. Transcripts were considered differentially expressed with a corrected *p*-value of <0.05, and log_2_ fold change > 1 or <1.

To accurately annotate the authentic start and stop codons of ORFs, rather than just the longest ORF, we used a modified version of TranSuite [8]. The original version of TranSuite is available here: https://github.com/anonconda/TranSuite (accessed on 15 May 2025). Our modified version of TranSuite is available here: https://github.com/mojtabagherian/TranSuite (accessed on 10 June 2025). Our modified TransFeat module creates a table (CSV) that lists common features related to NMD per transcript, such as distances to the last exon junction downstream of the stop codon and 3′ UTR length. If there was a downstream exon junction and it was at least 50 nucleotides after the stop codon, the transcript isoform is labelled as a Premature Termination Codon downstream Exon-Junction (PTC_dEJ_). Briefly, TranSuite first identifies the longest ORF in each transcript of the transcriptome provided [8]. Then TranSuite groups transcripts at the gene level, and start codons between transcripts to find the one that leads to the longest protein made by this gene [8]. This start codon leading to the longest protein from the gene is then used as the authentic start codon for all transcripts, even if that start codon does not lead to the longest ORF from that particular transcript [8]. The TranSuite algorithm does not explicitly search for Kozak sequence, the effect of secondary structure in the 5’ UTR, the ability of proteins to bind to elements in the 5′ UTR, or upstream ORFs when detecting the most authentic start codon.

The Kozak consensus sequence is the preferred sequence for eukaryotic translation initiation. We defined a match to a Kozak consensus sequence as the pattern RNNATGGV (where R = A or G and V = A or C or G). We searched the transcripts that changed ORF annotation between Reference Araport11 and Revised Araport11 with a Python3 script kozak.py, which is available on GitHub at https://github.com/mojtabagherian/TranSuite-Kozak (accessed on 28 September 2025).

To correctly annotate the Reference Araport11 transcriptome ORFs with PTC_dEJ_ presence/absence, we needed to filter out transcript IDs from the annotation file (GTF) that were missing from the Revised Araport11 transcriptome ORFs (TransFix output), to allow for a like-for-like comparison. The modified TransFeat was then used to identify whether a transcript had a PTC_dEJ_ or not. Because many transcripts in Araport11 are annotated with the longest ORF, and internal methionine codons would generate a longer ORF than using the authentic start codon, the stop codon and 3′ UTR does not change in Araport11—Reference ORF annotations.

Protein sequences were extracted from the default TransFeat module output table (last column, “Translation”) generated by TranSuite analysis. The protein sequences for RS2—Z33.1/PTB1.1 Reference ORF, RS2Z33.2/PTB1.2 Reference ORF, and RS2Z33.2/PTB1.2 Revised ORF were submitted to the AlphaFold 3 Server (https://alphafoldserver.com/, Google DeepMind) for structure prediction [33]. The highest-ranked models (model_0.cif files) were downloaded and visualised using UCSF ChimeraX (v.1.9). Structures were rendered with the RS2Z33.2/PTB1.2 Reference ORF shown in blue and the RS2Z33.2/PTB1.2 Revised ORF shown in purple to highlight the dramatic structural differences between the longest ORF and the biologically correct ORF predictions. Protein domains were annotated by SMART (http://smart.embl-heidelberg.de/) [34]. Linear protein sequence images were generated by Snapgene (https://www.snapgene.com/).

## 3. Results

**Figure 1:**
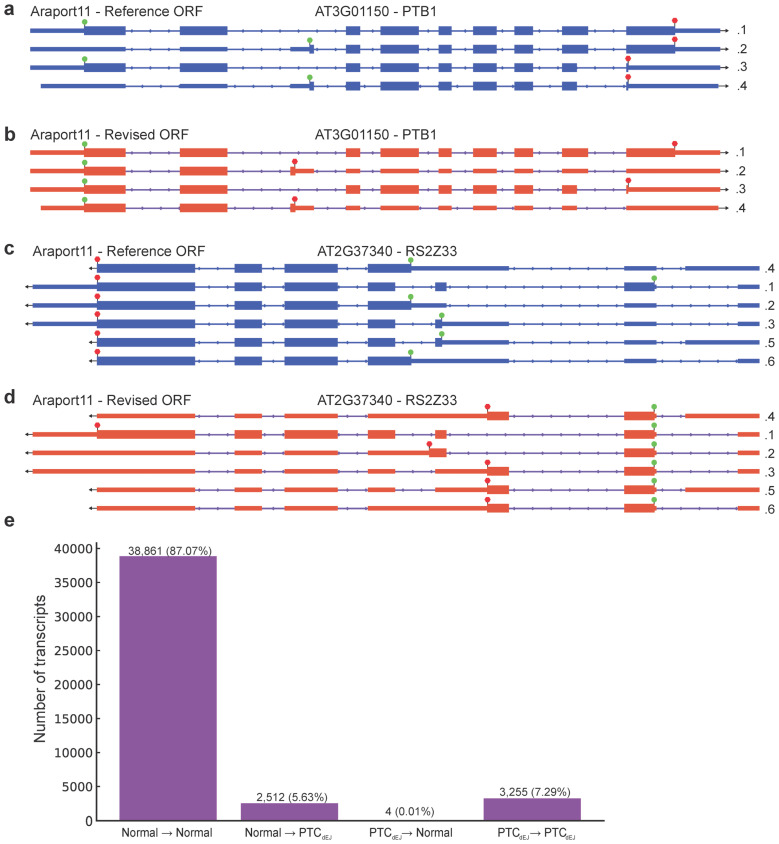
Araport11 transcripts with ORF annotations. (**a**) Araport11—Reference ORF annotations of AT3G01150 (*PTB1*) gene. Arrow shows the direction of the gene. The green circle indicates the start codon, the red octagon indicates the stop codon, and transcript expression level in transcripts per million (TPM) in wild type replicate 1 is shown next to each isoform model. Same in (**b**,**c**). PTB1 is on the forward strand of the TAIR10 genome. (**b**) Araport11—Revised ORF annotations of AT3G01150 (*PTB1*) gene. (**c**) Araport11—Reference ORF annotations of AT2G37340 (*RS2Z33*) gene. *RS2Z33* is on the forward strand of the TAIR10 genome. (**d**) Araport11—Revised ORF annotations of AT2G37340 (*RS2Z33*) gene. (**e**) Change in stop codon status from Araport11 Reference to Araport11 Revised. Normal stop to normal stop (38,861), normal stop to PTC_dEJ_ (2512), PTC_dEJ_ to normal stop 4), and PTC_dEJ_ to PTC_dEJ_ (3255).

The plant model *A. thaliana* transcriptome annotation Araport11 [5] has numerous examples of the longest ORF being selected for alternatively spliced transcripts (Figure 1). This leads to the appearance of a normal stop codon with an unmodified 3′UTR, but a large untranslated 5′ UTR (Figure 1). Identifying the longest ORF is computationally simple and logical but ignores the biological reality: The ribosome cannot predict which ORF is the longest but instead selects the same start codon as if the downstream AS event had not occurred. By annotating the longest ORF through using an internal methionine codon as the new start codon, the 3′ UTR of the AS-NMD target will look normal and the protein will appear as if has truncation of the N-terminal (Figure 1a,c). In reality, the protein would have a truncation at the C-terminal and the 3′ UTR would be much longer (Figure 1b,d), and likely contain at least one dEJ. Detection of the altered 3′ UTR can be used to computationally predict a transcript’s NMD-sensitivity [11,22,23,24].

We took the TranSuite software (modified) [8], developed to annotate the authentic start codon and stop codon of a transcript, and compared the Araport11 [5] transcriptome’s base annotations (Reference) to the TranSuite’s updated annotations (Revised). The known NMD targets *PTB1* and *RS2Z33* with Araport11 Reference ORF annotations have normal 3′ UTRs (Figure 1a,b), but once annotated with TranSuite, the presence of PTCs as defined by the presence of a dEJ at least 50 nt after the stop codon (PTC_dEJ_) is clear (Figure 1c,d). Therefore, the Araport11 Revised ORFs generated by TranSuite for these known NMD targets allows for easy detection of the NMD triggering features in these transcripts, while the Araport11 Reference ORFs appear to have a normal 3′ UTR and would go undetected (Figure 1). Over two thousand transcripts changed from a normal stop codon to a PTC_dEJ_ after TranSuite has been used, while only four go from PTC_dEJ_ to normal stop codon (Figure 1e). This striking asymmetry likely reflects that TranSuite correctly identifies the authentic start codon that would be used by the ribosome, while the Reference annotation artificially selects downstream methionines that create longer ORFs. Importantly, the transcript structure itself (exons and splice junctions) remains unchanged between Reference and Revised annotations; what changes is solely the computational definition of the ORF. While Araport11 selects the longest possible ORF per transcript, often using an internal methionine codon downstream of the authentic start site, TranSuite identifies the biologically relevant start codon that would be used consistently across all isoforms of a gene. The ribosome initiates translation at the first suitable AUG in the appropriate sequence context, regardless of any downstream splicing events that might create premature termination. Thus, when alternative splicing introduces a PTC, the Reference annotation artificially shifts to a downstream methionine to maximise ORF length, while the Revised annotation maintains the authentic start position that reflects actual translation initiation.

In order to determine the improvement from TranSuite ORF detection on Araport11 transcripts at the transcriptome-wide scale, we first analysed whether the change in predicted start codon led to a change in the likelihood that the start codon was more likely to be used for translation initiation. The Kozak consensus sequence has been identified in eukaryotes as a preferred sequence for translation initiation. Thus, we searched the transcripts with a change in ORF prediction (2516 transcripts; Figure 1e) for a change in start codon context and found that for the Revised Araport11 ORFs, 32% (805/2516) matched the Kozak consensus sequenced, compared to only 14% (363/2516) for the Reference Araport11 ORFs. This indicates that redefining the ORFs with TranSuite led to an increased rate of the start codon being in the preferred translation initiation context, and therefore a likely translon, rather than just a computationally predicted ORF.

To further validate if redefining ORFs improved the functional use of the transcriptome, we compared the Revised ORF with the Reference ORF annotations and the frequency of NMD target identification in an NMD-deficient mutant [31]. Specifically we looked at transcript isoforms with increased steady-state expression between the wild-type control and the *upf1 upf3* double mutant, which has reduced NMD pathway efficiency [31]. After NMD inhibition, direct targets of NMD are expected to increase in expression; however, many transcripts increased and decreased as a result of indirect effect resulting from the loss of NMD. In *A. thaliana*, mutations decreasing NMD activity result in indirect changes in pathogen response [35,36,37]. So, we expect only a small subset of changing transcripts to be direct targets, which is usually linked to NMD targeting features, such as a long 3′ UTR or downstream exon junction (PTC_dEJ_).

**Figure 2:**
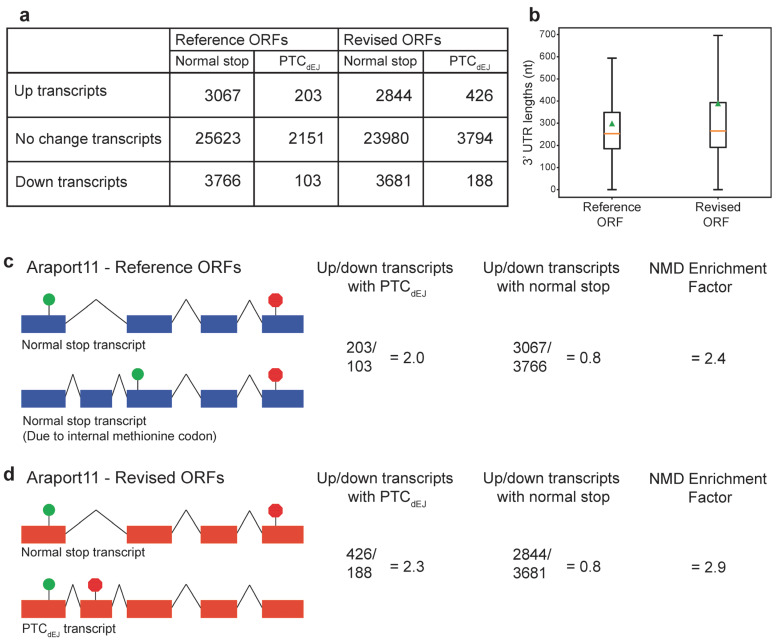
Prediction of authentic start-stop codons improves assessment of Araport11 transcripts. (**a**) Table indicating the number of up (increased steady state expression), no change, and down (decreased steady state expression) with normal stop codons and PTC_dEJ_ in our transcriptomic re-analysis. (**b**) Boxplot (outliers removed) of 3′ UTR lengths (nucleotides; nt) of up (increased steady state expression) transcripts in the NMD-deficient mutant, compared between the Araport11 Reference and Revised ORF annotations. Orange line represents the median. Green triangle represents the mean. Wilcoxon test *p* = 1.11 × 10^−41^, *n* = 3270, difference in mean = 91 nt. (**c**) Araport11—Reference ORF annotations and effect on NMD analysis. The number and ratio of up and down transcripts (steady state transcript levels) is shown with either PTC_dEJ_ or normal stop codons. The NMD enrichment factor is the ratio of up/down PTC_dEJ_ transcripts over the ratio of up/down of normal stop transcripts. The higher the number, the higher the enrichment of PTC_dEJ_ changing as predicted based on the EJC model of NMD and not transcripts with normal stop codons. Green circles represent start codons and red octagons represent stop codons. (**d**) Araport11—Revised ORF annotations and effect on NMD analysis. See description of (**c**) for details. Statistical comparison of the ratio of up/down PTC_dEJ_ transcripts in Reference and Revised ORF annotations was performed with Fisher’s exact test *p* = 0.37 so is not significant.

We then compared the fraction of putative NMD targeted transcripts (increased steady state expression) with NMD features with the Araport11 Reference ORF annotations or the Araport11 Revised ORF annotations, as reported by us here using our modified TranSuite [8]. We found that only 203 transcripts with increased steady state expression as annotated by Araport11 Reference ORF annotations had a PTC_dEJ_ (Figure 2a). In contrast, we found that 426 transcripts with increased steady state expression as annotated by Araport11 Revised ORF annotations had a PTC_dEJ_ (Figure 2a). Not all transcripts with PTC_dEJ_ are expected to be NMD targets; for example, many intron retained transcripts with PTC_dEJ_ are detained in the nucleus [38,39]. However, correct 3′ UTR annotation combined with increased steady state expression in an NMD-deficient mutant is highly indicative of an NMD target. Over two hundred transcripts would have been assumed to be indirect targets of NMD, due to the lack of an abnormal 3′ UTR if only the Reference ORFs had been considered (Figure 2a–d). Many past studies have reported that NMD targets have longer 3′ UTRs [11,22,23,24]. We confirmed that the increased steady-state expressed transcripts with the Revised ORF annotations had a longer average (mean) 3′ UTR than when the Reference ORF annotations were used (Figure 2b). Taken together, this indicates that our set of Revised ORF annotations is better at predicting the correct 3′ UTR structure of NMD targets.

Given that all transcripts with deceased steady-state expression will be indirect targets (trans-effects), we decided to use the ratio of increased (up) transcripts over the decreased (down) transcripts to assess the level of true NMD targets in that group. We predict that for a known NMD trigger (PTC_dEJ_), the ratio of up/down transcripts will be greater than one, with a higher ratio indicating more success at enriching for direct targets of NMD. We also predict that the ratio for transcripts with normal stop codons would be one or less. The ratio of up/down transcripts with PTC_dEJ_ is greater than one (2.0) when examining the Araport11 Reference ORF annotations (Figure 2c) and increases (2.3) when examining the Araport11 Revised ORF annotations (Figure 2b). This increase supports our notion that improving correct stop codon identification can help in identifying true NMD targets. In contrast, the ratio of up/down transcripts with normal stop codons remains largely unchanged (Figure 2c,d). TranSuite has globally identified more PTC_dEJ_-containing transcripts, not just those that are up. This is to be expected given the systemic mis-annotation of ORFs. The biological importance is not in selective enrichment, but in correct annotation finally revealing the true landscape of NMD targets, as validated by known examples and significantly longer 3′ UTRs.

**Figure 3:**
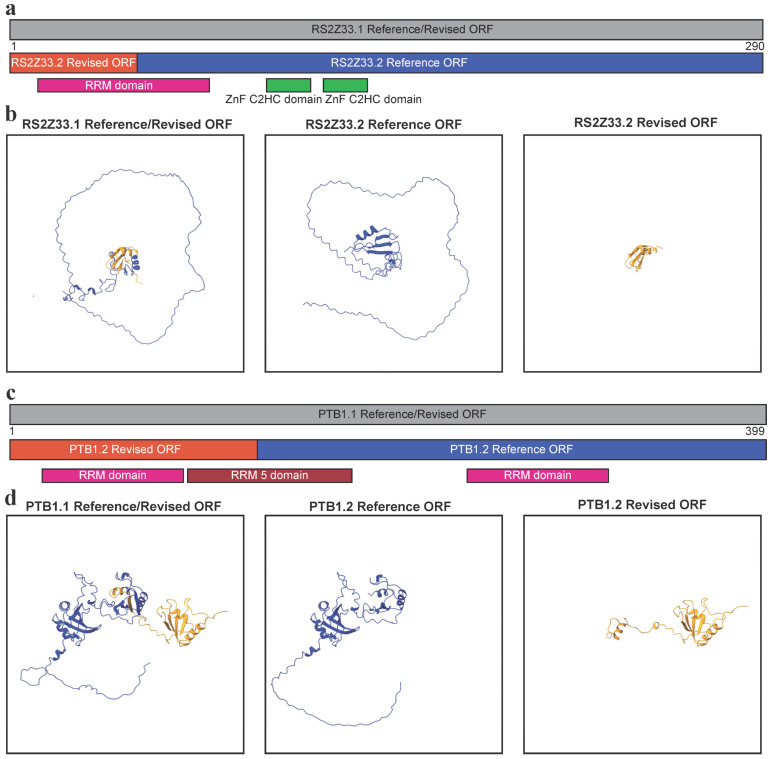
Impact of ORF annotation on protein structure predictions. (**a**) The RS2Z33.1 protein sequence with regions of RS2Z33.1 Reference and Revised ORFs that overlap. Domains are annotated. Image generated from Snapgene. (**b**) AlphaFold3-predicted protein structures of RS2Z33 isoforms. Left: RS2Z33.1 (AT2G37340.1) full-length protein. Middle: RS2Z33.2 (AT2G37340.2) Reference ORF—the incorrect long downstream ORF from Araport11. Right: RS2Z33.2 Revised ORF—the correct truncated protein predicted by TranSuite, resulting from intron retention and premature termination of translation. Yellow represents the protein sequence translated in .2 isoform as predicted by Revised ORF annotation, while blue represents the protein sequence translated in .2 isoform as predicted by Reference ORF annotation. (**c**) The PTB1.1 protein sequence with regions of PTB1.1 Reference and Revised ORFs that overlap. Domains are annotated. Image generated from Snapgene. (**d**) AlphaFold3-predicted protein structures of PTB1 isoforms. Left: PTB1.1 (AT1G01150.1) full-length protein. Middle: PTB1.2 (AT1G01150.2) Reference ORF—the incorrect long downstream ORF from Araport11. Right: PTB1.2 Revised ORF—the correct truncated protein predicted by TranSuite, resulting from alternative acceptor site splicing and premature termination of translation. Yellow represents the protein sequence translated in .2 isoform as predicted by Revised ORF annotation, while blue represents the protein sequence translated in .2 isoform as predicted by Reference ORF annotation.

Computational prediction of protein structure is becoming routine and automated [40], raising the concern that incorrectly annotated ORFs may pollute the databases with the structural predictions of incorrect proteins that are likely to never be translated and have no biological impact on the organism. It has been shown in the human disease facioscapulohumeral muscular dystrophy, that the natural loss of NMD through *DUX4* overexpression leads to the production of truncated proteins normally inhibited by NMD activity [41]. As these truncated proteins accumulate, they have a negative effect on cell viability through gain of function activity, as demonstrated by the overexpression of the human SR splicing factor *SRSF3* gene [41]. If the wrong ORF annotation was used to characterise the predicted truncated protein, misinterpretation would cloud these results. To demonstrate the potential severity of this, we used AlphaFold3 [33] to predict the structure of a plant SR splicing factor RS2ZZ33 (Figure 1b,d) and PTB1 (Figure 1a,c). Extracting the predicted protein sequence from the *RS2Z33.1* transcript isoform yields an identical amino acid string between the Araport11 Reference and Revised ORFs. However, for the intron retained *RS2Z33.2*, the introduction of an early stop codon leads to the aforementioned difference in ORF length (Figure 1b,d). When looking at the protein sequence, both the length and coding capabilities of *RS2Z33.2* are radically different (Figure 3a), with the Reference sequence encoding a protein of 260 amino acid residues and containing the second half of the RNA-binding RRM domain and two ZnF C2HC domains (Figure 3b). In contrast, the *RS2Z33.2* Revised sequence only encodes a short 55 amino acid residue long protein, which contains the first half of the RRM domain and no other known domains (Figure 3a). When AlphaFold3 [33] was used to generate structures for the RS2Z33.1 protein, a large protein with alpha helices and beta sheets is generated, with various intrinsically disordered regions (Figure 3b). A similar structure is produced from RS2Z33.2 Reference protein sequence, but some of the structured N-terminus is missing (Figure 3b). However, the RS2Z33.2 Reference protein sequence is very short and is mostly structured parts of the RRM domain (Figure 3b). When examining the coding potential of *PTB1* transcript isoforms, a similar picture emerged, with the *PTB1.2* (NMD targeted) isoform Reference and Revised ORFs differ in domain composition (Figure 3c). The full-length protein, PTB1.1, contains two RRM domains, separated by an RRM 5 domain (Figure 3c,d). The predicted Reference protein isoform PTB1.2 contains only the first RRM domain and half of the RRM 5 domain (Figure 3c,d). The predicted Revised protein isoform PTB1.2 also contains a single RRM domain and half of the RRM 5 domain, but not the second RRM domain (Figure 3c,d). Collectively, our findings show that protein structure predictions based on computationally derived longest ORFs, rather than biologically relevant ORFs, produce inaccurate structural models that risk contaminating databases and misleading future experimental design.

## 4. Discussion

Not all ORFs are translated [3]. ORFs are computational predictions of what could be translated within a given genome, but does not indicate whether they are translated by the ribosome [3]. Regions that are translated have been called translons to make the distinction between regions of potential protein coding and those that are translated by the ribosome [3]. Many translons are not ORFs, as they can use non-canonical start codons or ribosomal frameshifting that escape traditional ORF detection approaches [3]. Here, we are using an improved approach to identifying putative translons with the TranSuite software (modified) [8] to identify the start codon in each transcript most likely to be used for translation initiation, rather than just the longest ORF. The longest ORF is computationally simple to identify but unlikely to be a real translon. The approach we present here will still not capture all the true translons of a given transcriptome. This is because we did not focus on upstream ORFs, rare downstream ORFs, ribosomal frameshifting or selenocysteine. However, our goal was to better capture the main ORF [4] of each transcript: The primary translon. By performing this, we could better capture NMD targets (Figure 2) and predict relevant protein structures (Figure 3). To capture translons more fully, ribosome profiling will be needed [3]. However, ribosome profiling at the level of transcript isoforms is challenging [42], thus is limiting for detection of AS-NMD targets and the transcript features (long 3′ UTR and presence of a dEJ) associated with NMD targets.

In this study, we have shown that simply annotating the longest ORF does not exclusively reflect the biological reality of transcript anatomy, and that by annotating the biologically active (authentic) start codon, we can see an improvement in fraction of putative NMD identification (Figure 2). Not only is this important for studying NMD, but it can impact our predictions of protein structures in the proteome. If the reference ORF is used for in silico translation and protein structure prediction, the wrong conclusions about the nature of the truncated proteins would be drawn (Figure 3). These untranslated downstream ORFs would lead to computational hallucinations and should be purged from such databases of protein structure. Therefore, not simply annotating the longest ORF of a eukaryotic transcript but starting with a biologically relevant start codon can have a great benefit for that organism’s genomic resources. Transcripts can also be targets of NMD via upstream ORFs, which are short ORFs before the start codon of the main ORF. Our study has focused on the importance of selecting the correct start codon for the main ORF. Without selection of the correct main ORF, the short main ORFs of *PTB1* and *RS2Z33* may have been wrongly annotated as upstream ORFs.

Predicting direct NMD targets is challenging, as any method to inhibit NMD will have secondary effects on gene expression beyond preventing degradation of direct NMD targets. In *A. thalaiana*, many of these indirect targets of NMD are transcriptionally increased transcripts associated with the pathogen response [35,36,37]. Therefore, to examine the rules for what is recognised as an NMD target within a given organism, great care is needed. As direct NMD targets are predicted to increase after NMD inhibition, the number of decreased transcripts could give an indication of the expected trans-effects NMD target predictions. These trans-effects can result in transcript changes due to secondary effects of the loss of NMD factors from either the change in a direct NMD target causing transcriptomic changes, or due to NMD factors being involved in other cellular processes. We suggest that the ratio of up/down transcripts with a predicted NMD target signal (PTC_dEJ_) and those not expected to be NMD targets (normal stop codons) could be used to assess the level of NMD inhibition and describe this as the NMD enrichment factor (Figure 2). The PTC_dEJ_ is a well-known trigger of NMD in plants and animals [7], but when determining if this is a trigger in other eukaryotes, or looking to see if other transcript features are also NMD triggers, it is important to accurately annotate ORFs within the transcriptome. By using TranSuite [8], with our modified output (this study), not only can the accurate ORF be determined, but the presence of a PTC_dEJ_ or a long 3′ UTR can be immediately assessed. This will aid cross-species examination of the triggers of NMD.

There are many ways to assess the likelihood that a transcript is an NMD target or not. One highly accurate method is to assess the decay rates of transcripts in wild type and an NMD mutant. Briefly, if the decay rate of a transcript is less in the NMD mutant relative to wild type, the transcript is highly likely to be a direct NMD target. But studies looking at decay rates have focused on individual transcripts and not at the transcriptome-wide scale [35,43]. As previously mentioned, trans-effects resulting from inhibition of NMD in an organism eliminates the possibility of simply classifying all transcripts that are increased in an NMD mutant relative to wild type as an NMD target. But combining steady-state expression with known NMD triggers has been used by many to assess NMD targets in diverse eukaryotes [7,24,31]. Also, not all transcripts with these triggers are destined for degradation and can instead escape NMD. This can be due to proteins binding to long 3′ UTRs to prevent UPF1 binding [44] or because the transcript is never exported from the nucleus in a process termed “nuclear detention” due to the presence of a retained intron [38,39].

As long-read sequencing becomes cheaper, more accurate and more accessible, more high-quality genome assemblies will require annotations. Long-read sequencing of cDNA or direct RNA sequencing will also allow for improved annotations of the transcripts. However, correct ORF identification is still going to be a bottleneck here. By using TranSuite [8] to annotate the biologically accurate ORF, high quality predictions of the protein sequences can be brought in line with the improved quality of genome assemblies and transcript models of modern day genome projects. Already, the *A. thaliana* Reference Transcript Dataset 3 (AtRTD3) [45] is taking advantage of long-read sequencing of transcripts to greatly improve the transcriptome of *A. thaliana*, and is already taking advantage of TranSuite for accurate ORF predictions. Davis et al. (2025) recently used direct long-read RNA-seq in barley to identify novel transcripts and splicing events during pathogen infection, and benchmarked the best tools for structural annotation of transcript models [46]. Their excellent work demonstrated the importance of accurate transcript model annotations from long-read informed data. Complementing these transcript-level improvements, accurate ORF annotation represents an equally important consideration for reliable downstream interpretation. Our study outlines a simple improvement to computational annotation of bespoke transcriptomes generated by many studies that avoid the pitfall of simply annotating the longest ORF.

TranSuite overcomes the need for expensive and computationally intensive analyses of ribosome profiling to identify the biologically authentic start codon for a given transcript. In the future, it will be interesting to see if the ORFs predicted by TranSuite agree with those predicted by ribosome profiling or if improvements can be made to TranSuite’s approach by incorporating other predictive data, such as ribosome profiling or Kozak consensus sequence scoring, to ensure that the switching in start codon is appropriate. But when annotating the transcriptome of a recently sequenced eukaryote, or for annotating the transcriptome after new RNA-seq was performed and a bespoke transcriptome was generated with software such as StringTie [47], we highly recommend to researchers to use TranSuite rather than software that will just find the longest ORF per transcript.

## 5. Conclusions

Once a genome is sequenced, accurate annotation of the transcripts is needed, which can be assisted by RNA-seq to define the transcriptional start and end sites, and internal splice sites. However, accurate ORF annotation can be overlooked, leading to mis-annotation of start and stop codons that would generate the longest ORF within a transcript, but overlooks the biologically activated start codon within that transcript. Software tools such as TranSuite [8] overcome this limitation and allow for more accurate annotations of ORFs. Here, we have highlighted the importance of accurate ORF selection using the Araport11 annotation [5] of the model flowering plant *A. thaliana*, incorporating the biologically relevant start codon (Figure 1), we not only improve prediction of NMD targeted transcripts (Figure 2), but also of computational protein structure predictions (Figure 3). Improved ORF annotation will benefit any newly sequenced/annotated genome and any study that uses a bespoke annotation in their data analysis.

## Data Availability

A Zenodo archive with genomic data is available here: https://doi.org/10.5281/zenodo.15628154. It contains files used to run TranSuite (Athaliana_447_Araport11.gtf, Athaliana_transcripts.fa), intermediary output from TransFind/TransFix (Transfix.gtf), and the output from our custom TransFeat (Athaliana_transfeat_splice_junctions.csv). It also contains the modified Araport11 transcriptome (Athaliana_447_Araport11_Reference_ORF.gtf) used as direct input into TransFeat that was used to generate the Reference ORF annotations used in this study (Athaliana_transfeat_splice_junctions_Reference_ORF.csv). The Python3 script kozak.py is also available on GitHub; The original version of TranSuite is available here: https://github.com/anonconda/TranSuite (accessed on 15 May 2025). Our modified version of TranSuite is available here: https://github.com/mojtabagherian/TranSuite (accessed on 10 June 2025); For differential testing, data was downloaded from the NCBI Short Read Archive. SRR584118 (*upf1 upf3* replicate 1), SRR584124 (*upf1 upf3* replicate 2), SRR584115 (wild-type replicate 1), and SRR584121 (wild-type replicate 2).
